# Ammonia sets limit to life and alters physiology independently of pH in *Halomonas meridiana*

**DOI:** 10.1038/s41598-025-03858-z

**Published:** 2025-06-04

**Authors:** Cassie M. Hopton, Peter Nienow, Charles S. Cockell

**Affiliations:** 1 UK Centre for Astrobiology, School of Physics and Astronomy, University of Edinburgh, James Clerk Maxwell Building, Peter Guthrie Tait Road, Edinburgh, EH9 3FD UK; 2 School of Geosciences, University of Edinburgh, Drummond St, Edinburgh, EH8 9XP UK

**Keywords:** Astrobiology, Bacterial physiology, Bacteriology, Metabolomics

## Abstract

The subsurface oceans of icy moons, expected to retain appreciable concentrations of ammonia, are of significant interest to astrobiology. On Earth, ammonia is released in large quantities, primarily through anthropogenic activities. Ammonia is toxic to many forms of life at high concentrations, and thus it is necessary to understand the habitability impact of ammonia on these environments. The survival limits and physiological response of aerobic bacteria in ammonia, and whether ammonia toxicity is distinct from toxicity by high pH, is poorly understood. Here, we investigate the survival thresholds, growth kinetics, and metabolomic response of *Halomonas meridiana* in ammonia-water solutions and pH-matched sodium hydroxide solutions. Using closed- and open-air systems to mimic environments with NH_3_ retention or dispersion, we found complete and partial cell death above 0.05 M ammonia, respectively. In open-air systems, a sub-set of cells survived up to 0.25 M ammonia; metabolomics revealed unique physiological responses to ammonia, including elevation of cyclic compounds and Coenzyme A metabolites, suggesting mechanisms of ammonia toxicity and adaptation. Ammonia and high pH toxicity were found to be distinct. These findings show that ammonia can impose a distinct geobiological limit, potentially constraining the habitability of ammonia-rich terrestrial and extraterrestrial environments.

**Supplementary Information:**

The online version contains supplementary material available at 10.1038/s41598-025-03858-z.

## Introduction

Ammonia (hereafter considered as total ammonia, ammonia (NH_3_) + ammonium (NH_4_^+^)) is a primordial molecule, formed in the early universe and found abundantly in various environments including Earth’s early atmosphere and on other celestial bodies within the solar system^1–3^. Terrestrially, ammonia plays a critical role in the synthesis of amino acids and nucleic acids^4,5^, and has an integral role in the global nitrogen cycle^6,7^. Yet, despite biochemical significance, the toxicity of NH_3_ has been well-documented across the domains of life^8–11^. NH_3_ is gaseous under temperate conditions. The small size and unchanged nature of NH_3_ facilitates passive permeation through membranes^12–15^, causing significant intracellular disruption^16–18^. The equilibrium between NH_3_ and NH_4_^+^ is influenced by factors such as pH (with NH_3_ predominating at pH > 9.25), salinity and temperature^19–21^.

Mono Lake, California, underscores the role of NH_3_ in shaping microbial ecosystems; depth-dependent shifts in biodiversity are driven by the spatial distribution of abiotic factors including ammonia, which is predominately in the NH_3_ form^22,23^. This becomes relevant when considering the habitability of NH_3_-rich environments. Ammonia has been detected at a volume mixing ratio of 0.4–1.3% on the icy moon Enceladus, orbiting Saturn^24,25^. The evidence of a saline, liquid water subsurface ocean^26,27^, and other physicochemical conditions suitable for life^28–30^, has encouraged strong astrobiological interest in this satellite. With a pH predicted at or above 9^29–31^, the ocean of Enceladus would bear appreciable amounts of toxic NH_3_. Due to the limited research on NH_3_ tolerance in bacteria, coupled with the presence of carbon dioxide and hydrogen in the ocean, habitability assessments of Enceladus are currently limited to methanogenic organisms^32–34^.

The habitability impacts of ammonia are also relevant terrestrially. Globally, several dozen tetragrams of ammonia are released annually from agriculture^35,36^. Ammonia deposition into soil, water and vegetation occurs as a consequence^37–39^. In alkaline soils (pH > 7), application of ammonium fertilizers leads to NH_3_ volatilization. Indeed, applied nitrogen losses of up to 66% have been recorded in alkaline soils as a result of ammonium fertilizer application^40–42^. Although toxicological effects are presumed, the downstream impact of NH_3_ volatilization on microbial diversity and community structures in alkaline environments, where the relative abundance of NH_3_ exceeds NH_4_^+^, is not well understood.

To accurately assess the habitability impacts of ammonia, fundamental questions remain. Few studies have systematically investigated the survival limits or physiology of microorganisms under NH_3_ stress, where both the concentration and relative abundance of NH_3_ is high. Bacteria such as *Bacillus pasteurii* can grow in up to 0.5 M NH_3_^43^, although it is noted this species can utilise ammonia for ATP generation^44^, substrate permeability^45^ and oxidation of substrates^46^. Ammonia-oxidising bacteria (AOB) can metabolise both NH_3_ and NH_4_^+^ and can survive in up to 0.5 M ammonia at pH 6^47,48^, but the concentration of NH_3_ in these solutions is minimal (NH_3_ ≈ 0.05%, ≤ 0.00025 M). Without specific adaptations, toxicity at 0.1 M ammonia (pH 9.5, NH_3_ > 50%) has been observed in *B. subtilis* and *Escherichia coli*. However, similar toxicity was noted in sodium chloride solutions of matching pH^49^. The use of neutrophilic organisms has thus far limited our understanding of whether ammonia toxicity is intrinsically pH-based. There is compelling need for research examining the survival limits and physiological response of an alkaline adapted bacteria in ammonia solutions exceeding pH 9.25 (NH_3_ > 50%) and comparing with a pH matched counterpart.

Here, we use a combination of growth kinetics and cell viability assays to identify habitability limits of *Halomonas meridiana* Slfth1 in ammonia solutions with high NH_3_ content. This organism lacks specific adaptations to ammonia^50^, but possesses alkalitolerant traits relevant for this investigation^51^. We utilise growth systems permitting (“open-air system”) or preventing (“closed-air system”) gaseous escape to mimic environments where NH_3_ is environmentally dispersed and retained, respectively. By using an alkaline adapted organism, we resolve that ammonia toxicity is independent from pH toxicity and, using microscopy and metabolomics analysis, define the specific toxicity effects of ammonia on bacterial physiology independent of pH. Together, these findings advance the understanding of ammonia toxicity and should foster improved habitability assessments of NH_3_-rich environments.

## Results

### Physicochemical properties of ammonia and pH-matched solutions

**Fig. 1:**
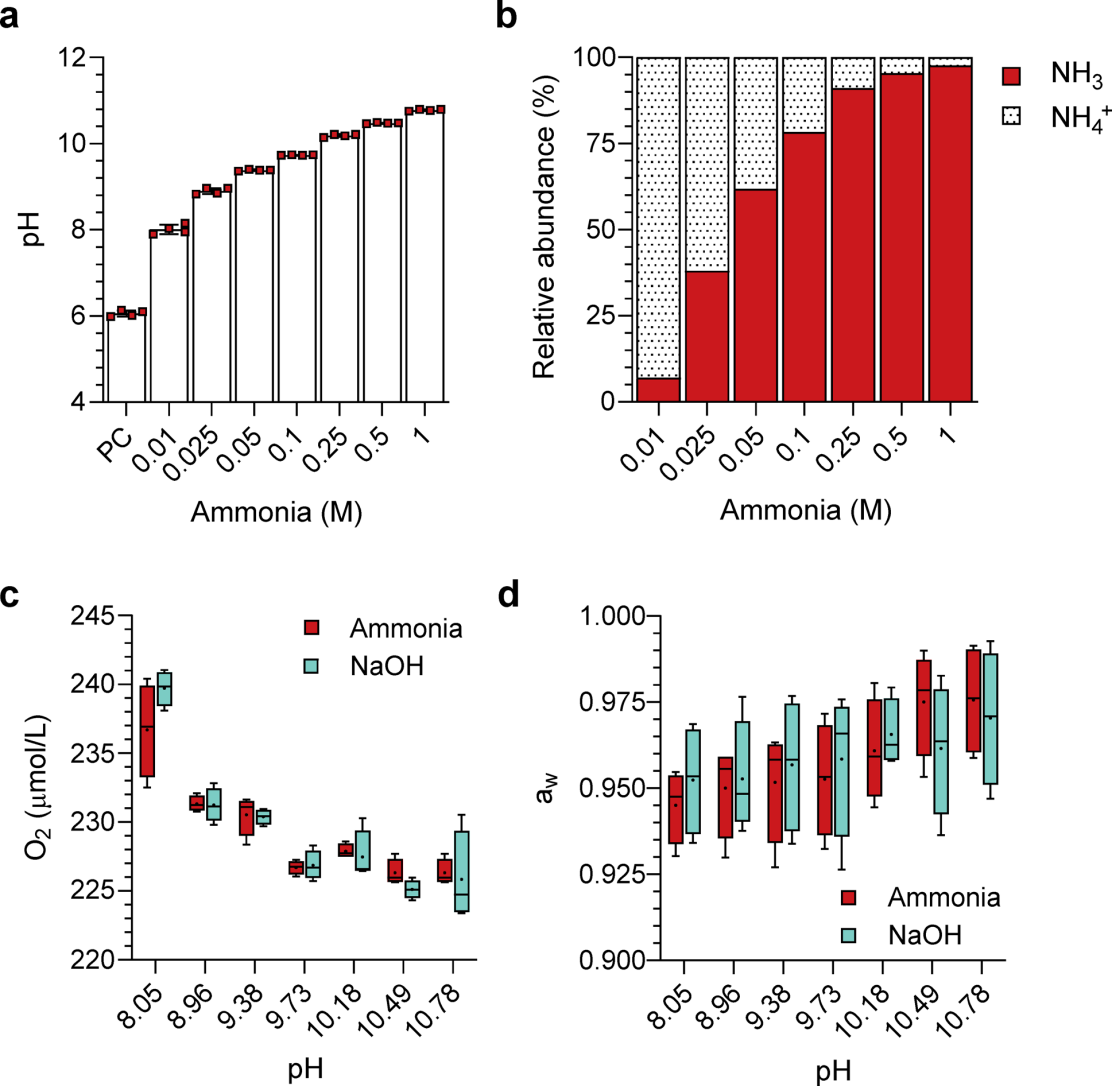
Properties of ammonia and pH-matched solutions utilised in this study. (**a**) Molar levels of ammonia and corresponding pH values utilised in this study. The positive control (PC) corresponds to growth in unamended yeast media (0 M ammonia) without pH modifications. Column heights and error bars represent mean ± s.d. (*n* = 4). (**b**) Calculated percentage abundance of NH_3_/NH_4_^+^ in molar levels of ammonia utilised in this study. (**c**) Oxygen concentration and (**d**) water activity of ammonia and pH-matched solutions utilised in this study. The upper limit, middle line and lower limit of the boxplots indicate the 25th, 50th (median) and 75th percentiles, respectively. Whiskers represent 1.5× the interquartile ranges. Mean is indicated by a plus sign (+) (*n* = 4). Alterations to oxygen concentration and water activity were found to be non-significant between all ammonia and pH-matched counterparts. Significance is given by unpaired two-tailed t-tests or Mann-Whitney test at each pH. The statistical tests and outcomes are available in Supplementary Table 1.

Ammonia solutions were prepared from liquid ammonia to molar concentrations of 0.01, 0.025, 0.05, 0.1, 0.25, 0.5 and 1 M, equating to pH values between 8 and 11 (Fig. 1a). Positive control (PC) was unamended yeast media. The percentage abundance of NH_3_ to NH_4_^+^ at these concentrations and pH values is given in Fig. 1b. From concentrations of 0.05 M ammonia and higher, NH_3_ accounts for over 50% of the total NH_3_/NH_4_^+^ in solution. Solutions of increasing ammonia concentration showed decreased oxygen concentrations; however, this decrease was non-significant from pH-matched counterparts (Fig. 1c). Alterations to water activity with increasing ammonia and pH were also statistically non-significant (Fig. 1d).

### Ammonia sets a distinct molarity threshold for viability and growth

**Fig. 2:**
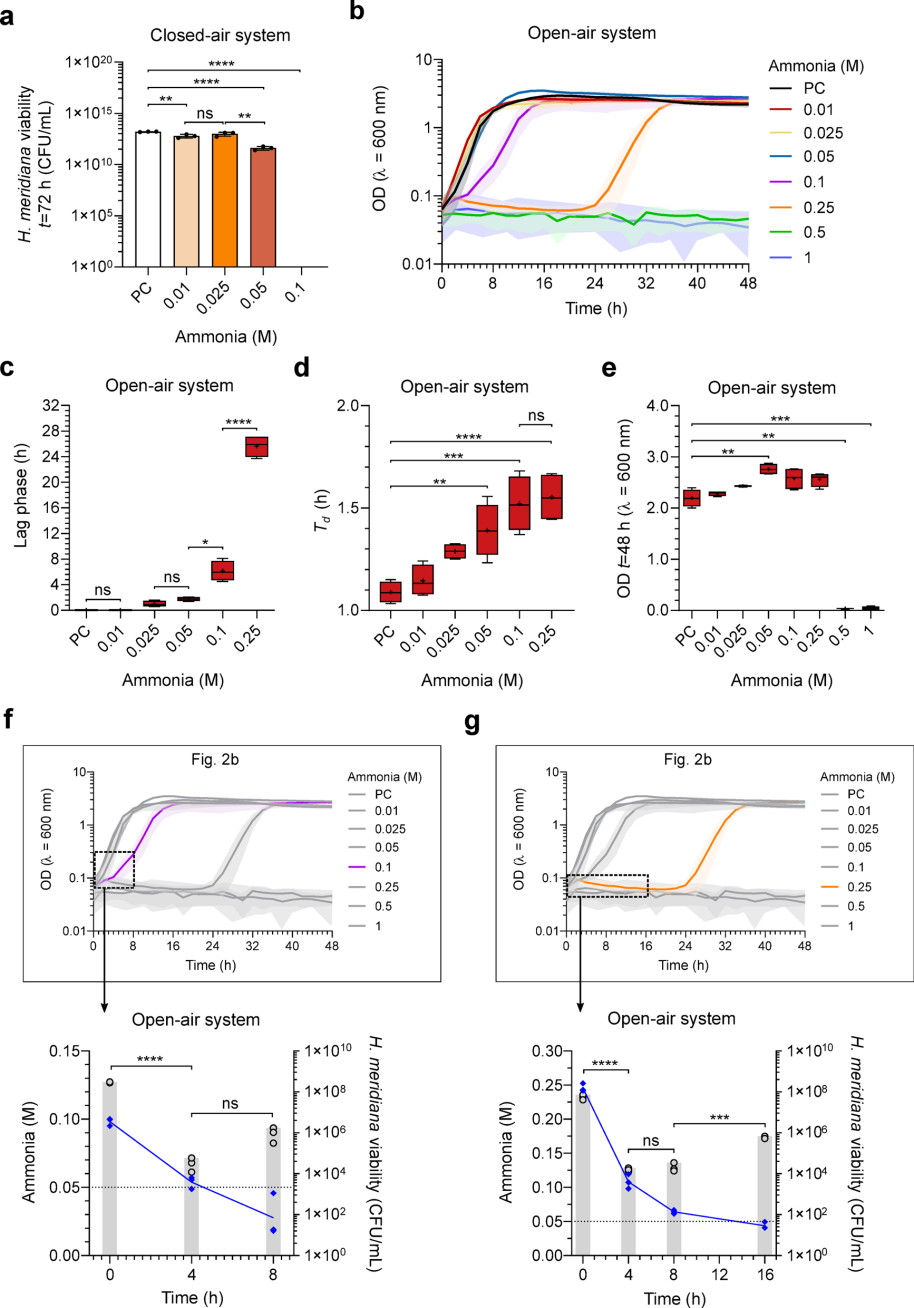
Survival limits of *H. meridiana* in ammonia. (**a**) CFU/mL of *H. meridiana* grown in ammonia solutions at 0 (PC), 0.01, 0.025, 0.05 and 0.1 M in a closed system for 72 h. The positive control (PC) corresponds to growth in unamended yeast media (0 M ammonia) without pH modifications. Column heights and error bars represent mean ± s.d. (*n* = 3). Statistical significance is by one-way ANOVA with Tukey’s multiple comparison test (*F* (4, 10) = 27.70, *p* < 0.0001). (**b**) Growth curve of *H. meridiana* over 48 h in increasing ammonia concentrations. Growth curves represent mean OD_600_ values over time ± s.d. (0-0.5 M, *n* = 4; 1 M, *n* = 3). Error is indicated by area fill within error bands. Growth parameters of lag phase (**c**), doubling time (*T*_*d*_) (**d**) and final OD_600_ at 48 h (**e**) extrapolated from (**b**) are presented. Box plots represent the median as well as the 25% and 75% interquartile ranges. The whiskers represent 1.5× the interquartile ranges. Plus sign (+) indicate the mean and the middle line indicate the median. Welch’s ANOVA with Games-Howell’s multiple comparison test was used in (**c**) *W* (3.000, 5.965) = 218.6, *p* < 0.0001) and (**e**) *W* (7.000, 9.736) = 7919, *p* < 0.0001). Statistics in (**d**) correspond to one-way ANOVA with Tukey’s multiple comparison test (*F* (5, 18) = 14.79, *p* < 0.0001). (**f**, **g**) Time series of ammonia molarity and cell viability (CFU/mL) during the lag phase and early growth of cultures grown in 0.1 (**f**) and 0.25 M ammonia (**g**). The relevance to data points in Fig. 2b is indicated. Ammonia molarity is plotted as a line graph with blue diamond markers on the left axis. Molarity at 0.05 M is indicated by a dotted line. Cell viability is plotted as a column bar graph on the right axis. Line and column heights represent mean ± s.d. (*n* = 3). For cell viability, statistical difference between means is given by unpaired *t*-test. ns, no significance; *, *p* < 0.05; **, *p* < 0.01; ***, *p* < 0.001; ****, *p* < 0.0001.

Bacteria were grown in ammonia solutions utilising two system types: a ‘closed-air’ system – to determine growth limits in a perpetually ammonia exposed environment, and an ‘open-air’ system – to determine growth limits in an environment permitting NH_3_ dispersal following volatilization. Figure 2a shows the final colony forming units of *H. meridiana* after incubation in the closed-air system for 72 h. The closed-air system utilised an air-tight falcon tube for culture that was presumed to maintain constant ammonia concentrations by retention of both aqueous and volatilized ammonia. Colonies were evident following incubation in 0.01 M, 0.025 M and 0.05 M ammonia, but colony number decreased as NH_3_ increased relative to NH_4_^+^ (Fig. 1b). A lower number of viable colonies compared to positive control was observed in 0.01 M ammonia (*p* < 0.01). No significant difference in colony number was observed when *H. meridiana* was incubated in 0.025 M compared to positive control (*p* = 0.056) or 0.01 M ammonia (*p* = 0.323). This concentration of ammonia has a pH within the optimum range for *H. meridiana* (pH 9) (Fig. 1a). Lower viable cell numbers were observed when incubated in 0.05 M compared to positive control (*p* < 0.0001) and 0.025 M ammonia (*p* < 0.01). No viable colonies were present following incubation in 0.1 M ammonia or ammonia solutions of higher concentration (Fig. 2a).

Figure 2b shows the growth dynamics of *H. meridiana* Slthf1 over 48 h in increasing concentrations of ammonia in an open-air system. Lag phase duration, doubling time (*T*_d_) and final OD_600_ are presented in Fig. 2c-e, respectively, extrapolated from Fig. 2b of the open-air system. At concentrations exceeding 0.01 M ammonia, lag phase was greater with higher ammonia concentrations; incubation in 0.1 M and 0.25 M ammonia prolongs lag phase time by 6-fold and 25-fold compared to the positive control, respectively (Fig. 2c). The lag phase time in 0.1 M ammonia was higher than that in 0.05 M ammonia (*p* < 0.05). Likewise, the lag phase time in 0.25 M ammonia was higher than that in 0.1 M ammonia (*p* < 0.0001) (Fig. 2c). *T*_*d*_ was also higher with increasing ammonia concentration, with higher *T*_*d*_ compared to the positive control observed in 0.05 M (*p* < 0.05), 0.1 M (*p* ≤ 0.0001) and 0.25 M (*p* < 0.0001) solutions (Fig. 2d). The *T*_*d*_ was not significantly altered between positive control and 0.01 M (*p* = 0.965) or 0.1 M and 0.25 M solutions (*p* = 0.9972). Final OD_600_ after 48 h growth showed no statistical difference from positive control in 0.01 M (*p* = 0.932), 0.025 M (*p* = 0.330), 0.1 M (*p* = 0.248) and 0.25 M solutions (*p* = 0.135), but higher OD_600_ values were observed in 0.05 M compared to the positive control (*p* < 0.01) (Fig. 2e). Cell density was lower in 0.5 and 1 M solutions compared to the positive control (0.5 M, *p* < 0.01; 1 M, *p* < 0.001) (Fig. 2e), where no distinguishable growth occurred within 48 h (Fig. 2b).

In Fig. 2b, ammonia appeared to extend lag phase in 0.1 M and 0.25 M ammonia cultures. Onset of the log phase following extended lag phase could indicate an inhibitory, bacteriostatic influence of ammonia that had diminished over time possibly due to NH_3_ volatilization and dispersal from the culture solution. However, as the optical density at the lag phase (< 0.05) was below the detectable limit, it is possible cell death events occurring during this period were not adequately represented in the data. Thus, a bactericidal effect whereby cell numbers were reduced and gradually repopulated to numbers that were detectable by optical density could not be ruled out. To deconvolute bacteriostatic and bactericidal effects of ammonia, cell viability within lag phase and early log phase relevant time points were performed in 0.1 M and 0.25 M ammonia culture solutions. Relevant time points align to 0, 4 and 8 h for 0.1 M ammonia solutions, and 0, 4, 8, and 16 h for 0.25 M ammonia solutions. Additionally, ammonia concentrations were measured over these time points to identify whether NH_3_ volatilization and dispersal correlated with growth initiation. The results for 0.1 M and 0.25 M cultures are presented in Fig. 2f and 2g, respectively. After 4 h exposure, viable cells in 0.1 M ammonia showed a 1000-fold decrease from 0 h (*t* = 28.28, df = 4, *p* < 0.0001) (Fig. 2f). In 0.25 M ammonia, viable cells at 4 h were reduced 10-fold from 0 h (*t* = 6.499, df = 4, *p* < 0.01) (Fig. 2g). Bactericidal reduction to cell populations within 0.1 M ammonia solutions cease between 4 h and 8 h, where a small but non-significant increase in cell viability was observed (*t* = 2.421, df = 4, *p* = 0.0727) (Fig. 2f). Likewise, bactericidal effects were absent between 4 h and 8 h in 0.25 M ammonia solutions where cell numbers stabilised (*t* = 0.8934, df = 4, *p* = 0.442) and increased from 8 h to 16 h (*t* = 11.76, df = 4, *p* < 0.001) (Fig. 2g). The increase in cell viability within 0.1 and 0.25 M ammonia solutions at 8 h and 16 h, respectively, aligned with diminished ammonia levels to ≤ 0.05 M at 4 h and 13 h, respectively (Fig. 2f, g). Thus, lag phase extension in the open-air system reflects two events: (1) an immediate bactericidal effect and (2) growth initiation after ammonia levels drop to sub-bactericidal levels (≤ 0.05 M) in surviving populations.

### Ammonia toxicity is independent from pH toxicity

**Fig. 3:**
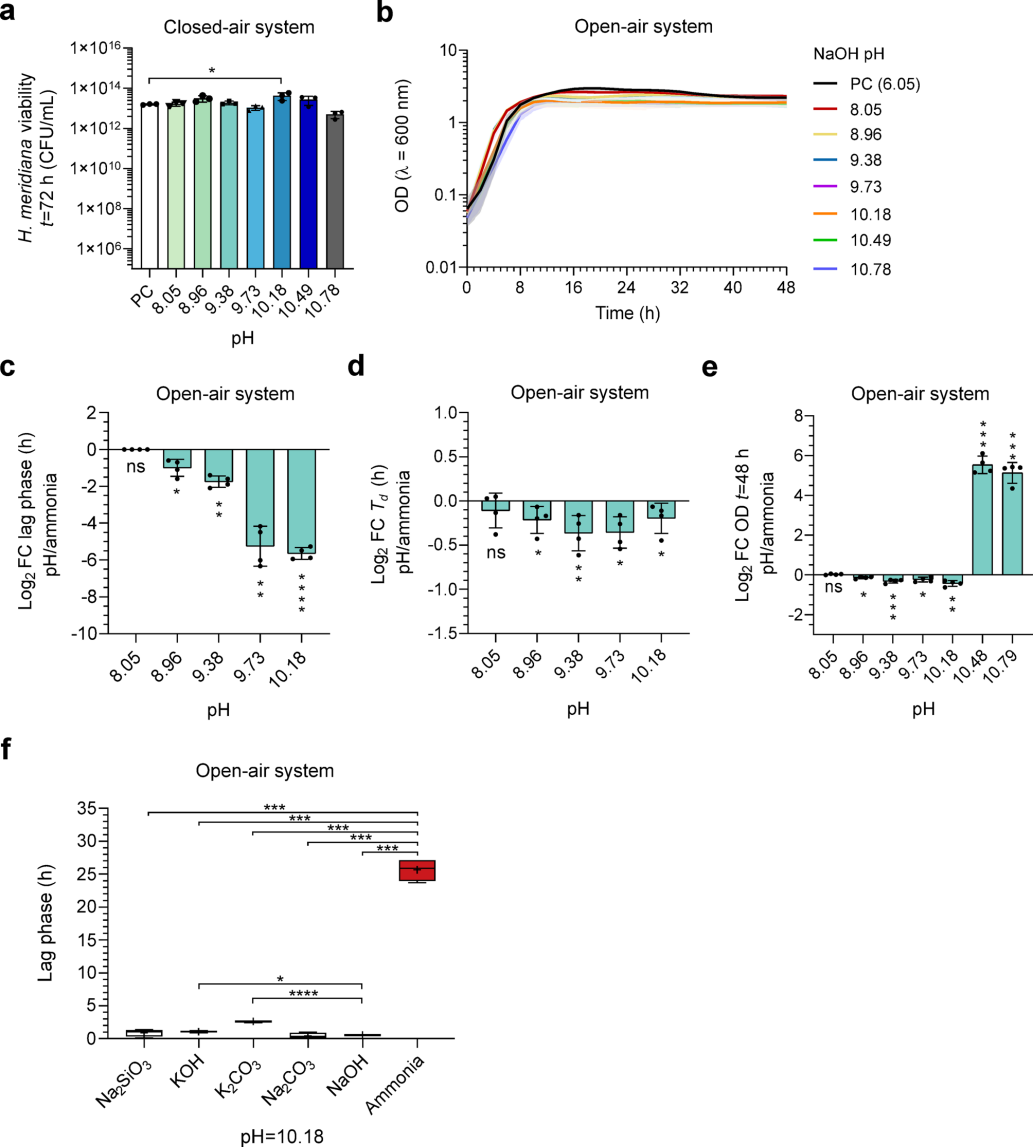
Growth of *H. meridiana* in NaOH pH-matched solutions. (**a**) CFU/mL of *H. meridiana* grown in a closed system for 72 h with solutions of increasing pH that pH-match ammonia solutions of 0 (PC, pH 6.06), 0.01 (pH 8.05), 0.025 (pH 8.96), 0.05 (pH 9.38), 0.1 (pH 9.73), 0.25 (pH 10.18). 0.5 (pH 10.49) and 1 M (pH 10.78). The positive control (PC) corresponds to growth in unamended yeast media (0 M ammonia) without pH modifications. Column heights and error bars represent mean ± s.d. (*n* = 3). Statistical significance is by one-way ANOVA with Tukey’s multiple comparison test (**F** (7, 16) = 5.292, *p* < 0.01). (**b**) Growth curve of *H. meridiana* over 48 h grown in NaOH solutions of increasing pH. Growth curves represent mean OD_600_ values over time ± s.d. (*n* = 4). Error is indicated by area fill within error bands. Growth parameters of lag phase (**c**), doubling time (*T*_*d*_) (**d**) and final OD_600_ at 48 h (**e**) extrapolated from (**b**) are presented as Log_2_ fold change (FC) values comparing pH/ammonia. Statistics correspond to unpaired two-tailed t-tests and non-parametric tests comparing the data for lag phase, *T*_*d*_ and final OD_600_ at each pH condition to the corresponding ammonia molarity. Statistical tests are detailed in Supplementary Table 3. Column heights and error bars represent mean ± s.d. (*n* = 4). (**f**) Box plots comparing the lag phase of *H. meridiana* grown in different pH-matched solutions at pH 10.18. Box plots represent the median as well as the 25% and 75% interquartile ranges. The whiskers represent 1.5× the interquartile ranges. The plus signs (+) indicate the mean and the central line indicate the median (*n* = 4). Welch’s ANOVA with Games-Howell’s multiple comparison test was used (**W** (5.000, 8.089) = 170.4). ns, no significance; *, *p* < 0.05; **, *p* < 0.01; ***, *p* < 0.001; ****, *p* < 0.0001.

Ammonia is a weak base that raises solution pH with increasing concentration. To delimitate ammonia toxicity from pH toxicity, growth experiments were repeated in NaOH solutions pH-matched to ammonia solutions. Figure 3a presents the final CFU/mL of *H. meridiana* in pH-matched solutions following 72 h closed-air system incubation. No significant differences were observed between cells grown in positive control and pH-matched solutions up to pH 10.78 (equivalent to 1 M ammonia), with exception of cells grown in pH 10.18 where cell number was higher (*p* < 0.05). The outcome of the statistical analyses are available to view in Supplementary Table 2. Figure 3b presents the growth curve of *H. meridiana* grown under pH-matched NaOH solutions in an open-air system, with extrapolated parameters of lag phase (Fig. 3c), *T*_d_ (Fig. 3d) and final OD_600_ (Fig. 3e) presented in Log_2_ fold changes (Log_2_FC) from growth in ammonia solutions of the same pH. For cells grown in pH 8.05 and 0.01 M ammonia, there was a non-significant difference between lag phase duration (*U* = 8, *p* > 0.999), *T*_*d*_ (*t* = 1.55, df = 6, *p* = 0.171), and final OD_600_ (*t* = 0.798, df = 6, *p* = 0.456). But, significantly altered lag phases, *T*_*d*_ and final OD_600_ were observed for higher pH and ammonia values. Lower lag phases were observed in cells grown in pH 8.96, pH 9.38, pH 9.73 and pH 10.18 compared to those grown at 0.025 M (*t* = 4.399, df = 3, *p* < 0.05), 0.05 M (*t* = 11.26, df = 3, *p* < 0.01), 0.1 M (*t* = 7.663, df = 3.02, *p* < 0.01) and 0.25 M ammonia (*t* = 29.05, df = 3.05, *p* < 0.0001) (Fig. 3c). *T*_*d*_ was significantly lower for cells grown in pH 8.96 (*t* = 2.453, df = 6, *p* < 0.05), pH 9.38 (*t* = 4.235, df = 6, *p* < 0.01), pH 9.73 (*U* = 0, *p* < 0.05), and pH 10.18 (*t* = 2.532, df = 6, *p* < 0.05) compared to ammonia counterparts (Fig. 3d). Final OD_600_ at 48 h was lower in NaOH pH 8.96 (*t* = 4.577, df = 3, *p* < 0.05), pH 9.38 (*t* = 7.914, df = 6, *p* < 0.001), pH 9.73 (*t* = 2.786, df = 6, *p* < 0.05) and pH 10.18 (*t* = 5.173, df = 6, *p* < 0.01) than to growth in ammonia counterparts. Higher OD_600_ was observed in pH 10.49 (*t* = 16.09, df = 3.01, *p* < 0.001) and pH 10.78 solutions (*t* = 15.59, df = 3.12, *p* < 0.001) compared to ammonia counterparts (Fig. 3e), indicating lag phase extension and absent growth in 0.5 and 1 M ammonia could not be attributed to pH increases.

To broaden the pH comparison beyond NaOH, lag phase analysis was used to explore whether the prolonged lag phase in 0.25 M ammonia (Fig. 2c) could be replicated in KOH, Na_2_SiO_3_, K_2_CO_3_, and Na_2_CO_3_ at pH 10.18 (Fig. 3f). No differences in lag phase duration were observed between *H. meridiana* grown in Na_2_SiO_3_ (*p* = 0.779) or Na_2_CO_3_ (*p* = 0.996) compared to NaOH. Longer lag phases were seen in KOH (*p* < 0.05) and K_2_CO_3_ (*p* < 0.0001) compared to NaOH, possibly reflecting reduced adaptation to potassium in *H. meridiana*. All high pH cultures showed a significantly shorter lag phase than 0.25 M ammonia cultures (*p* < 0.001), confirming the pH-independent toxicity of ammonia.

### Ammonia toxicity exerts distinct changes to bacterial morphology

**Fig. 4:**
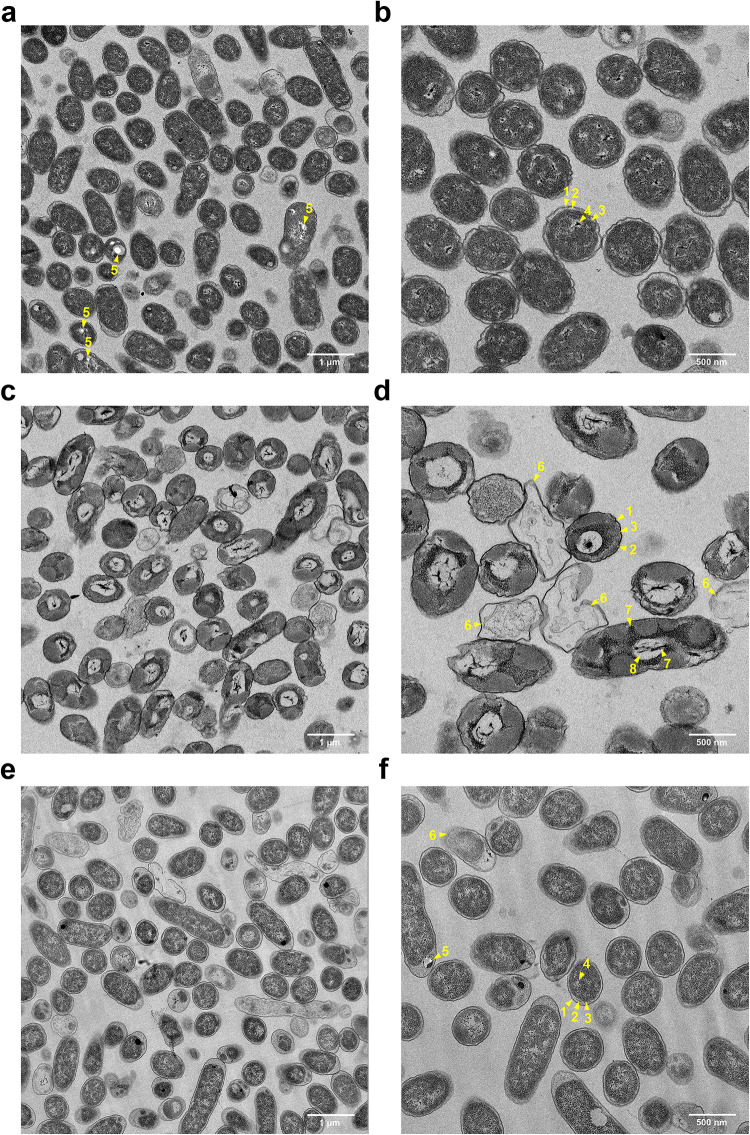
TEM of *H. meridiana*. *H. meridiana* after 2 h treatment in control solutions (**a**, **b**), 1 M ammonia (**c**, **d**), and pH-matched yeast media (pH 10.78, **e**, **f**). Yellow arrows: 1, outer membrane; 2, periplasmic space; 3, inner membrane; 4, nucleoid; 5, PHA-like granule; 6, lysed cell; 7, aggregated material; 8, electrolucent cavities.

Specific bactericidal effects of ammonia on cells can be observed in Fig. 4. Under control growth conditions (unaltered yeast media) (Fig. 4a-b), *H. meridiana* exhibited ribosome-rich cytoplasm, visible nucleoids and clear division of outer membrane, periplasm and inner membrane. Cells exhibited irregular, undulating outer membrane, an enlarged periplasmic space and PHA-like granules. Upon 2 h treatment of 1 M ammonia (Fig. 4c-d), cells showed intracellular aggregation and loss of ribosomes with some showing cytoplasmic loss and lysis. The periplasmic space volume decreased, and detachment of the inner membrane from the outer membrane was observed. Cells showed expansion of electrolucent cavities, possibly surrounding nucleoids. Condensed material within the electrolucent cavities exhibited splayed morphologies, possibly indicating disruption to DNA supercoiling. Cells exposed to a NaOH solutions pH-matched to 1 M ammonia (10.78 pH) also showed some cell lysis events (Fig. 4e-f). However, there were few morphological differences from cells grown in control media. Differences include uniform outer membrane, and fewer PHA-like granules.

### Metabolic pathways altered in response to ammonia and high pH exposure

**Fig. 5:**
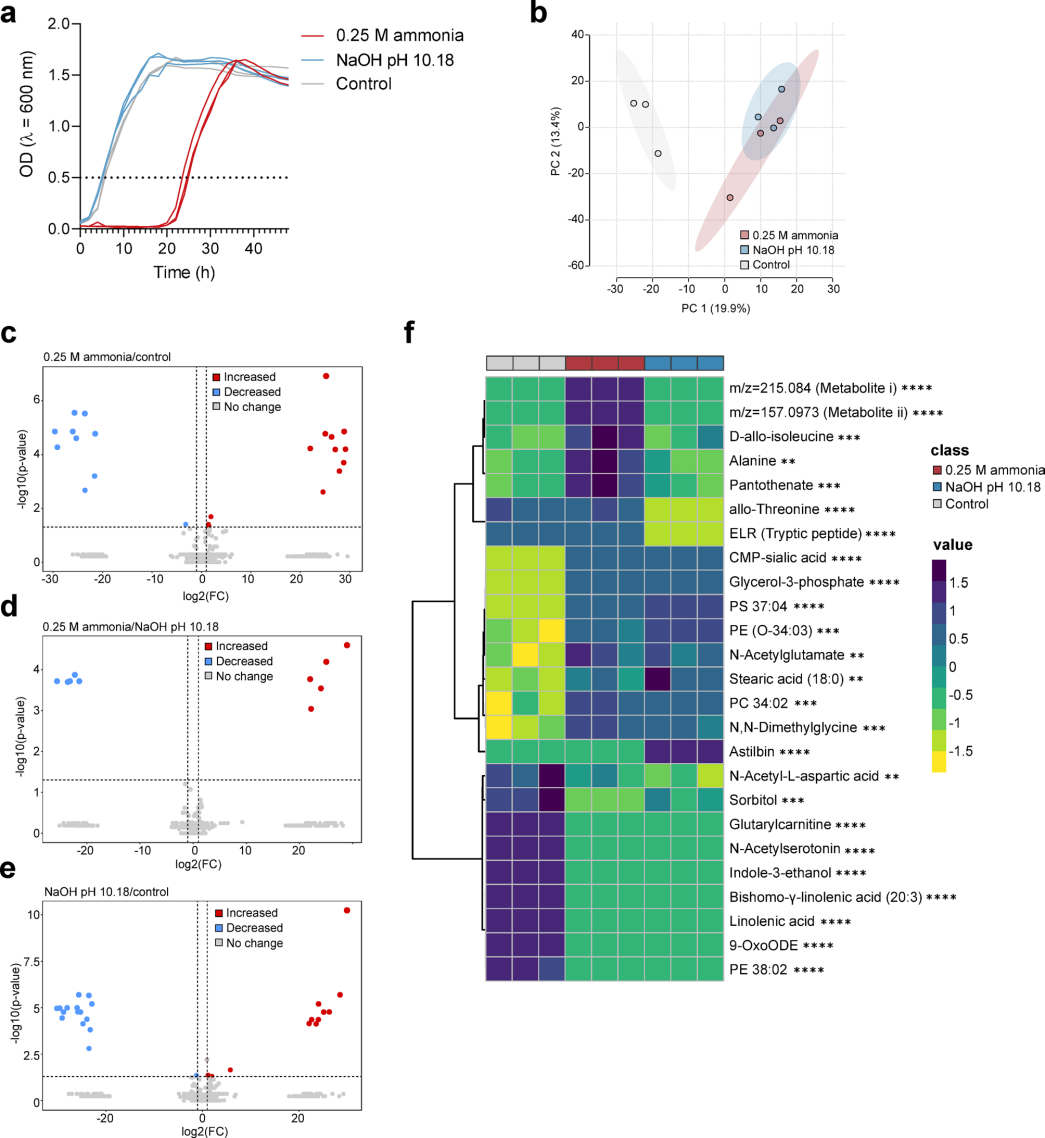
Metabolic features significantly altered upon ammonia and high pH exposure in *H. meridiana*. (**a**) Growth curve of samples cultured for metabolomics processing with each replicate shown. Dotted line at OD_600_ = 0.5 indicates harvest point. (**b**) Scores plot of a principal component analysis (PCA). (**c**–**e**) Volcano plots depicting metabolites with a fold change greater than 2 and a *p*-value lower than 0.05 (adjusted using FDR correction) for 0.25 M ammonia/control (**c**), 0.25 M ammonia/NaOH pH 10.18 (**d**) and NaOH pH 10.18/control (**e**). Relative levels of each metabolite are presented as red dots (high) or blue dots (low). (**f**) Heatmap of the top 25 metabolites most significantly altered between ammonia, pH and control treated samples. Significance was calculated by one-way ANOVA (alpha = 0.05, two-sided) with Tukey’s post-hoc test using Metaboanalyst 6.0. Significance level is indicated next to metabolites. The normalised relative abundance is presented in a gradient from dark blue (high) to yellow (low). All data compiled from three biological replicates (*n* = 3). The control corresponds to growth in unamended yeast media (0 M ammonia) without pH modifications. PCA, volcano plots and heatmap were generated using MetaboAnalyst 6.0 and amended for visual clarity in Inkscape. **, *p* < 0.01; ***, *p* < 0.001; ****, *p* < 0.0001.

The survival of cells in up to 0.25 M ammonia in an open-air system suggests adaptations enabling tolerance to ammonia. Untargeted metabolomics was performed on *H. meridiana* in unamended yeast media (hereafter denoted ‘control’) and yeast media with 0.25 M ammonia at pH 10.18 (hereafter denoted ‘0.25 M ammonia_’_) to determine variations in metabolism that may account for the differences observed. To delimitate high pH adaptations from ammonia adaptations, metabolomics was also performed following exposure to yeast media adjusted to pH 10.18 with NaOH (hereafter denoted ‘NaOH pH 10.18’). Growth kinetics and sampling points in these conditions are indicated in Fig. 5a. Principal component analysis (PCA) (Fig. 5b) separated 0.25 M ammonia and NaOH pH 10.18 conditions from the control, with overlap between 0.25 M ammonia and NaOH pH 10.18 conditions indicating metabolic similarity. Univariate volcano analysis identified 23 features significantly altered in 0.25 M ammonia/control (Fig. 5c), 10 features significantly altered in 0.25 M ammonia/NaOH pH 10.18 (Fig. 5d), and 28 features significantly altered in NaOH pH 10.18/control (Fig. 5e). The volcano analysis dataset for each comparison group is shown in Supplementary Tables 4–6.

The most significantly altered metabolites between these conditions were identified by ANOVA (Supplementary Table 7), depicted as a heatmap (Fig. 5f). Overall, high similarity was found between 0.25 M ammonia and NaOH pH 10.18 exposed samples; both conditions generally exhibited higher levels of unsaturated phospholipid and lower levels of linoleic acid and derivatives. Intermediates that feed glycerophospholipid biosynthesis, CMP-sialic acid (**F** = 2021.9, *p* < 0.0001) and glycerol-3-phosphate (**F** = 2022.2, *p* < 0.0001), were more abundant compared to the control. Amino acid pathway intermediates indole-3-ethanol (**F** = 2327.8, *p* < 0.0001), N, N-dimethylglycine (**F** = 30.626, *p* < 0.001) and N-acetylglutamate (**F** = 25.653, *p* < 0.01) were also altered compared to control. There were reduced levels of N-acetylserotonin (**F** = 18876, *p* < 0.0001) and N-acetyl-L-aspartic acid (**F** = 24.091, *p* < 0.01) that could suggest reactions with acetyl donors were less favourable at high pH in both conditions.

### Ammonia exposure elicits a unique metabolomic response

**Fig. 6:**
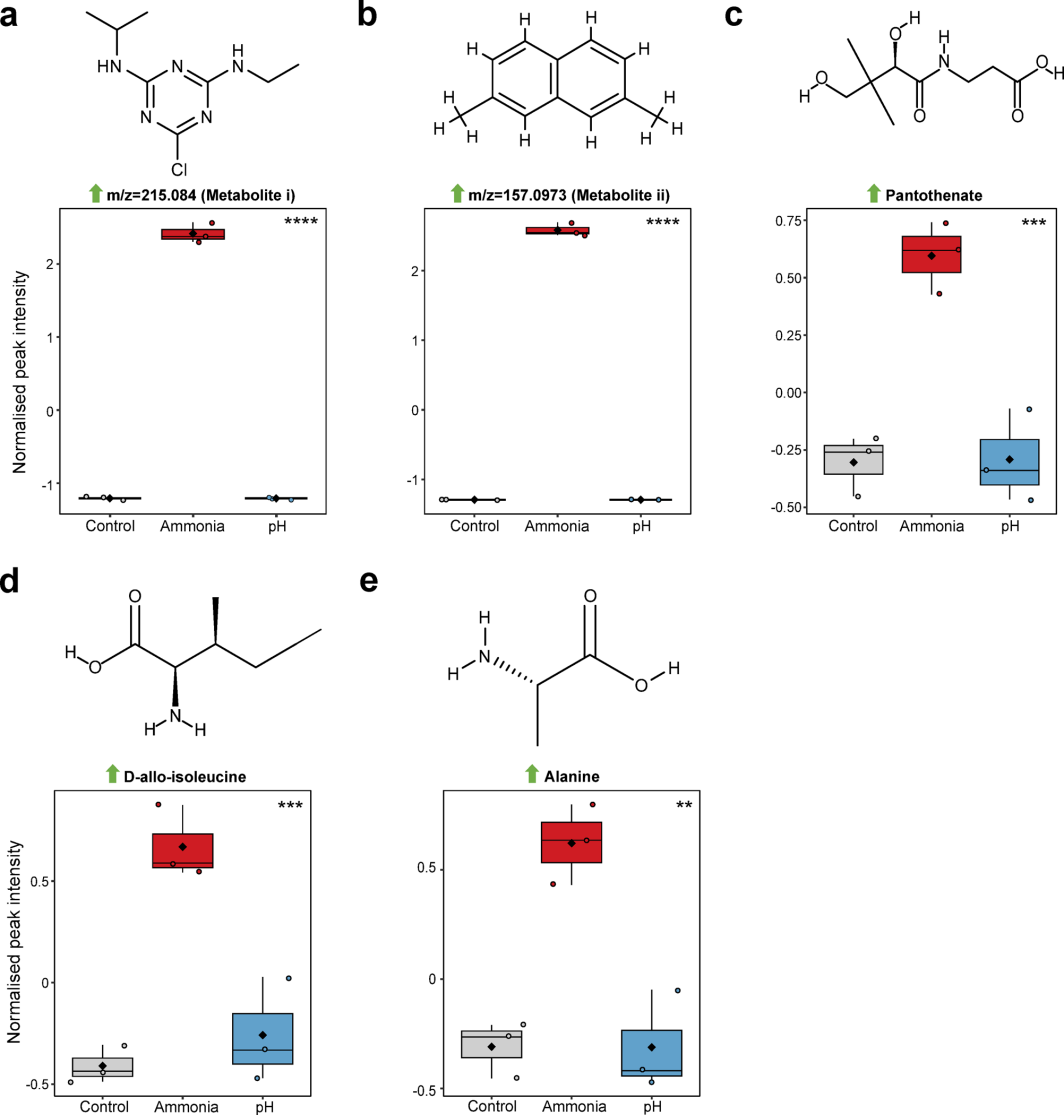
Metabolic response to ammonia exposure in *H. meridiana*. (**a**–**e**) Box plots of five significantly altered metabolites found in 0.25 M ammonia exposed *H. meridiana* (ammonia) compared to NaOH pH 10.18 (pH) and control samples (control). Results are taken from ANOVA with Tukey’s post-hoc test (Supplementary Table 7). The upper limit, middle line and lower limit of the boxplots indicate the 25th, 50th (median) and 75th percentiles, respectively. Whiskers represent 1.5× the interquartile ranges. Mean is indicated by a black diamond. Coloured circles represent the values from all samples (*n* = 3 for each group). Box and whiskers were generated using MetaboAnalyst 6.0 and edited for visual clarity in Inkscape. Chemical structures were created using ChemDraw. **, *p* < 0.01; ***, *p* < 0.001; ****, *p* < 0.0001.

Despite general similarities between cells exposed to 0.25 M ammonia and NaOH pH 10.18, ANOVA indicated five metabolites significantly altered only in 0.25 M ammonia cultured *H. meridiana*. Box plots of these metabolites are presented in Fig. 6a-e. Samples exposed to 0.25 M ammonia showed significantly higher levels of unidentified metabolites, metabolite i at m/z = 215.084 (Fig. 6a) and metabolite ii at m/z = 157.0973 (Fig. 6b), compared to NaOH pH 10.18 exposed samples (metabolite i, *p* < 0.0001; metabolite ii, *p* < 0.0001) and control samples (metabolite i, **F** = 2112.3, *p* < 0.0001; metabolite ii, **F** = 4865.6, *p* < 0.0001). Library annotations matched metabolite i to nitrogen-containing heterocyclic compound atrazine (89.6% Q score) and metabolite ii to hydrocarbon 2,7-dimethylnaphthalene (96.5% Q score). Structures of atrazine and 2,7-dimethylnaphthalene are depicted in Fig. 6a and b, respectively. Univariate analysis also revealed exposure to 0.25 M ammonia increased the relative abundance of pantothenate (**F** = 28.839, *p* < 0.001) (Fig. 6c), and amino acids D-allo-isoleucine (**F** = 28.686, *p* < 0.001) (Fig. 6d), and alanine (**F** = 25.391, *p* < 0.01) (Fig. 6e), compared to those in NaOH pH 10.18 and control conditions.

## Discussion

The influence of ammonia on habitability, particularly to microbes, is underrepresented in scientific literature. Yet, the pollution of ammonia into the environment could shape the underlying microbial community structures and biodiversity of alkaline environments. The presence of ammonia within the subsurface ocean of Enceladus, predicted at an alkaline pH, may also shape the habitability expectations of this satellite. Although the presence of life beyond Earth is speculative, terrestrial organisms can be utilised to explore the boundaries of known life under similar conditions to assess potential for habitability. In this study, we utilise alkalitolerant and halophilic *H. meridiana* as an analogue organism to investigate the molar concentration thresholds for survival, and physiological changes, that might be present in aerobic bacteria within extreme NH_3_ environments.

The exposure of *H. meridiana* to increasing ammonia concentrations revealed that growth at 0.05 M ammonia, where the relative abundance of NH_3_ is 62%, sets a consistent habitability limit for *H. meridiana* regardless of whether in a closed-air or open-air system. The tolerance of microbes to ammonia varies and cannot be predicted by genus or species alone; strain-specific responses to stress have been well documented^52–57^. The molarity limit established in this study aligns with data for other bacteria, including *B. subtilis* strains T1, T2, T19, T22, T33, T39^43^ and *Enterobacter cloaecae* HNR^16^, but is lower than the absolute limit for ammonia survival in literature which extends to above 0.716 M (NH_4_)_2_SO_4_ at pH 9, correlating to above 0.5 M NH_3_, survived by strains of *B. pumilus and B. pasteurii*^43^. Specific ammonia adaptations may support the survival of *B. pasteurii* in such concentrations^44–46^. The relative abundance of NH_3_ to NH_4_^+^ in these studies is below 40%. We define an NH_3_-rich solution as one where NH_3_ exceeds natural environmental concentrations for ammonia (> 6 ppm, assessed by the Agency for Toxic Substances and Disease Registry)^58^, and where the relative abundance of NH_3_ exceeds NH_4_^+^. Our results characterise a habitability limit of 0.05 M in an ammonia solution that can be considered NH_3_-rich by this definition (550 ppm, NH_3_/NH_4_^+^ = 62%), which has not been previously reported for an aerobic bacterium.

Independent ammonia and pH toxicity has been characterised in *E. coli*^49^, yet indistinguishable toxicity has been recorded with *B. subtilis*, *Sporosarcina*,* Paenibacillus*,* Staphylococcus*,* Brevibacillus*,* Streptomyces*,* Pseudomonas and Arthrobacter*^9,49^. Using an alkalitolerant organism, we establish ammonia toxicity is distinct from pH toxicity in *H. meridiana* and does not exert toxicity via oxygen displacement or water activity changes. However, the degree of similarly between the metabolomic profiles of cells exposed to 0.25 M ammonia and a pH-matched NaOH solution indicates *H. meridiana* primarily responds to ammonia using high pH adaptations. Unique physiological responses to ammonia in *H. meridiana* included the accumulation of two cyclic compounds, metabolite i and ii, the former of which bore amine functional groups. The presence of these compounds may be indicative of intracellular NH_3_-driven reactions^59,60^. The disruption of such structures to cellular components could be a mechanism of pH-independent toxicity. Indeed, *H. meridiana* treated with ammonia exhibited aggregation to intracellular components that would suggest significant disruption to cell contents. Reductions to free ammonia concentration coupled with formation of metabolites i and ii could correlate the formation of metabolites i and ii with intracellular NH_3_-driven reactions.

Elevated levels of alanine, D-allo-isoleucine, and pantothenate were also exclusively observed in ammonia exposed *H. meridiana.* The presence of an isoleucine derivative and pantothenate suggested modulation to the Coenzyme A pathway that affects the cell membrane^61,62^, and amino acid metabolism^62–64^. Indeed, D-amino acids have been implicated in cell wall remodelling in *Vibrio cholerae* and *B. subtilis*^65^. Alanine dehydrogenase catalyses the production of alanine by a reaction between pyruvate and ammonium^66–68^. Thus, alanine could be a by-product of elevated ammonia levels. Notably, D-amino acid aminotransferase catalyses the production of pyruvate and D-glutamate ​from D-alanine and 2-oxoglutarate, for which D-allo-isoleucine can act as an amino donor in *Thermotoga maritima*^69^. The presence of D-allo-isoleucine may therefore lead to enrichment of pyruvate and, in turn, enrichment of D-alanine or L-alanine via the alanine dehydrogenase pathway, or both. D-alanine may act to impede ammonia permeation; D-alanine can increase rigidity, and decrease permeability, of the peptidoglycan layer^70–72^. L-alanine may act to prevent excessive ammonia metabolism; in some bacteria, L-alanine is an allosteric inhibitor of the nitrogen assimilation enzyme glutamine synthetase^73–75^.

Interpreted from the point of view of planetary habitability, the closed-air system is the most applicable representation of icy moon subsurface oceans, as ammonia concentrations are presumed to remain constant. The ammonia toxicity limit at 0.05 M established in the closed-air system is higher than the lowest predicted concentration of ammonia at 0.01 M for the Enceladus ocean, but lower than the highest predicted concentration at 0.1 M^25,30,31^. When considering the proportion of more toxic NH_3_, the pKa of the NH_3_/NH_4_^+^ system can be presumed to increase as temperature decreases; a pKa of ~10.1 at 0 °C has been approximated^76^. The pKa of the NH_3_/NH_4_^+^ equilibrium is also influenced by ionic strength^21^. Thus, with oceanic temperatures estimated at 0 ºC^28^ and ionic strength at 0.333 molal^31^, it can be calculated that the Enceladus ocean would require a pH of < 10.6 to satisfy a relative abundance of NH_3_ at < 62% suggested as a habitability boundary in this sstudy. This is in the range of the lower pH estimations currently modelled for Enceladus, where phosphate speciation in the waters constrains the ocean to pH 10.1 to 11.6, with a most consistent value of pH 10.6^31^.

On Earth, up to 78 tetragrams of ammonia per annum are expelled globally from agricultural processes^35,36^. Individual livestock buildings show ammonia concentrations up to 30 ppm (0.002 M)^77,78^, which is lower than the habitability limit indicated in this study, yet, it should be noted a water volume of 1 L would require just 1 gram of ammonia to exceed an ammonia concentration of 0.05 M. In alkaline environments, this may alter bacterial community structures and biodiversity once the molarity and relative abundance of NH_3_ exceeds thresholds for survival, as has been observed in Mono Lake^22^. Bactericidal effects from NH_3_ may be transient, unless NH_3_ deposition is continuous, due to the open-air volatilization and dispersal of NH_3_ – repopulation of *H. meridiana* in an open-air ammonia system was observed at concentrations above 0.05 M ammonia. However, it is not without mention that localised and recurrent “point sources” of ammonia deposition have been identified in both agriculture and industrial processes that may consistently disturb bacterial habitats despite volatilization and dispersal^79^.

Without field-based environmental surveys, the effects of NH_3_ on terrestrial and extraterrestrial habitability are only estimated. Additionally, without isolation and biological characterisation of metabolites i and ii, we can only speculate the contribution of these molecules in ammonia toxicity. However, we have shown that bacterial growth and viability may become impacted when molar concentrations of ammonia are above 0.05 M, where NH_3_ exceeds NH_4_^+^. Moreover, we deduce that ammonia and pH toxicity are distinct, suggesting a need to modify the current understanding of NH_3_ toxicity in bacteria. Together, these findings can be integrated into the current knowledge regarding the impact of ammonia on terrestrial geomicrobiology and may inform future habitability assessments of NH_3_-rich extraterrestrial environments.

## Materials and methods

### Solution Preparation

Solutions of liquid ammonia were made to the required molar concentrations—0.01, 0.025, 0.05, 0.1, 0.25, 0.5, 1 M ammonia in yeast media using 35% NH_3_ (Fisher Scientific, CAS Number: 1336-21-6). The pH of the solutions was determined with a Jenway 3510 benchtop pH meter. pH-matched sodium hydroxide solutions (NaOH; Fisher Scientific, CAS Number: 1310-73-2) were prepared by gradual addition of 1 M NaOH into yeast media. To compare growth in different basic solutions at a pH equivalent to that of 0.25 M ammonia (pH 10.18), several bases were selected that have a natural pH at or greater than pH 10.18 and high solubility in water. Thus, yeast media was made to pH 10.18 ± 0.01 using gradual addition of 1 M NaOH, 1 M potassium hydroxide (KOH), 1 M sodium metasilicate (Na_2_SiO_3_), 1 M potassium carbonate (K_2_CO_3_) or 1 M sodium carbonate (Na_2_CO_3_). Growth experiments proceeded as outlined below. pH was matched to within ± 0.01 pH units. All solutions were filter-sterilised through a 0.22-micron pore before use.

### NH_3_/NH_4_^+^ percentage abundance

The percentage concentration of ammonia was determined by indirect calculation based on commonly utilised equations developed by Hampson^19^. Firstly, the ionic strength of the prepared solutions based on known salinity (0.2 M NaCl) was determined by Eq. 1.

1 $$\:I=19.9273\left(S\right)/(1000-1.005109\left(S\right))$$

Where I = molar ionic strength; S = salinity (parts per thousand, ppt).

The stoichiometric acid hydrolysis constant of ammonium ions, pKa^s^, based on I, was then calculated. pKa^s^ values for given ionic strengths were provided by Hampson^19^. These values were plotted to obtain a linear regression for calculating pKa (Supplementary Fig. 1). pKa^s^ was calculated from Eq. 2.

2 $$\:pK{a}^{s}=0.1179\left(I\right)+9.246$$

Percentage abundance of NH_3_ at given salinity, pH and temperature was then calculated from Eq. 3. As salinity, temperature, pressure and pKa^s^ are constant in the experiment, only pH was modified to give percentage abundance of NH_3_ at each pH value (Supplementary Table 8).

3 $$\%\:N{H}_{3}=100/[1+{10}^{\left({pKa}^{s}+0.0324\left(298-T\right)+0.0415\left(P/T\right)-pH\right)}]$$

Where *P* = 1 atm pressure; T = temperature (K).

### Oxygen measurements

Oxygen concentrations (µmol/L) of unamended yeast media, ammonia solutions and pH-matched solutions, prepared as described above, were recorded using an oxygen microsenser with tip diameter of < 2 mm (OX-500, Unisense, Denmark). Solutions were equilibrated for 0.5 h before reading at 28 ºC in 96-well plates sealed with aluminium foil to prevent gas loss.

### Water activity

Unamended yeast media, ammonia solutions and pH-matched solutions were prepared as described above. Solutions were equilibrated for 1.5 h and water activities measured at 28 ºC with a Rotronic HP23-AW water activity meter (Rotoronic AG, Bassersdorf, Switzerland).

### Bacterial strain selection

*Halomonas meridiana* Slthf1 (DSM 15724; Gram negative) was obtained from the German Collection of Microorganisms and Cell Cultures (DSMZ). The organism was originally isolated at a depth of 2000 m from low temperature hydrothermal fluid in the East Pacific Rise. The strain exhibits halophilic growth up to 22% NaCl, and shows tolerance up to pH 12^50^. The strain is therefore representative of the physiological attributes that could be necessary to grow in saline, alkaline fluids. A complete genome sequence is available for this strain^50^. This organism is not known to have specific adaptations to ammonia; this was a deliberate choice. The purpose of this study was not to study microbial biochemical adaptation to high ammonia, but rather to study whether NH_3_ can act as an abiotic factor influencing bacterial growth, survival and physiology.

### Bacterial culture

An aerobic culture of *H. meridiana* was grown in glass conical Erlenmeyer flasks at 28 ºC in an orbital benchtop shaking incubator set to rotate at 150 RPM. We assume ammonia-rich environments present optimal nutrient conditions (i.e., available organic debris) with a salinity < 2% for the purpose of this study. Thus, *H. meridiana* Slthf1 was grown in a yeast media consisting of 1 g/100 mL Bacto™ yeast extract (Becton, Dickinson and Company), 0.2 M NaCl (1.17% salinity) (Thermo Fisher Scientific, CAS Number: 7647-14-5) and distilled water (dH_2_O).

### Growth experiments

Two growth systems were implemented: ‘closed’ growth experiments were conducted in sealed air-tight 15 mL falcon tubes with sufficient headspace (14 mL) to allow aerobic metabolism but prevent ammonia evaporation; ‘open-air’ growth experiments were conducted in 96-well plates to permit ammonia evaporation and to monitor growth dynamics. Growth in closed-air system experiments were assessed by colony forming units (CFU) after 72 h incubation at 28 ºC and 150 RPM. Growth in open-air systems were assessed in 96-well plates by optical density (OD) readings in a BMG SPECTROstar Nano Microplate Reader at 600 nm with reading taken every half an hour for 48 h at 28 ºC. Plates were shaken at 200 RPM before each reading. Plate wells were prepared with 190 µL of a selected solution and seeded with 10 µL overnight *H. meridiana* culture to OD_600_ = 0.05. Positive control wells were 190 µL unamended yeast media inoculated with 10 µL overnight *H. meridiana* culture to OD_600_ = 0.05. Negative controls had no inoculation. To avoid condensation, plate lids were coated with Triton X-100 (0.05%) in 20% ethanol.

### Growth parameters

Experiments were conducted to yield three standard microbiological growth parameters: lag phase duration, doubling time (*T*_d_), and final OD_600_. The lag phase was calculated with a web-based microbial lag phase calculator^80^, with the following parameters: algorithm = parameter fitting to a model; pre-processing applied: cut data at some time = yes, max time = 24–48 h; smooth data = no; initial biomass = first observation; model to fit = logistic; NLS fitting algorithm = auto; max number of iterations = 1000. *T*_d_ was calculated by first determining the growth rate in the exponential growth phase (*µ*) as per Eq. 4, followed by calculation of *T*_d_ as per Eq. 5.

4 $$\:\mu\:=\left({Log}_{10}\left(N\right)-{Log}_{10}\left({N}_{0}\right)\right)2.303/(t-{t}_{0})$$

Where N_0_ = OD_600_ at the beginning of a selected time interval (*t*_*0*_) in the exponential growth phase; N = OD_600_ at the end of a selected time interval (*t*) in the exponential growth phase. *t* and *t*_*0*_ are recorded in minutes.

5 $$\it \:{T}_{\mathrm{d}}=ln2/\mu\:$$

The final OD_600_ reached after 48 h was recorded and used to represent the final cell concentration reached. Cell viability versus OD_600_ over 24 h was assessed by PrestoBlue^™^ cell viability reagent (Thermo Fisher Scientific, Massachusetts, USA). Calibration curves confirm changes to observed OD_600_ corresponds to increasing number of viable cells (Supplementary Fig. 2). Growth or lack of growth was determined by the presence or absence of a defined lag phase, exponential phase, and stationary phase within a 48 h growth period.

### Ammonia evaporation and cell viability

In separate 96-well plates, overnight culture of *H. meridiana* was inoculated to OD_600_ = 0.05 in 0.1 M and 0.25 ammonia solutions. Plates were incubated at 28 ºC on an orbital shaker continuously shaken at 150 RPM and tested as described below at 0, 4, and 8 h for 0.1 M ammonia solutions, and 0, 4, 8, and 16 h for 0.25 M ammonia solutions. Separate 96-well plates were used for each time point. The viability of cells in the culture wells at these time points was assessed by CFU on yeast media agar plates incubated at 28 ºC. Ammonia content of culture wells at these time intervals was recorded using the CHEMetrics High Range VACUette Ammonia test kit (K-1510 C) by direct nesslerization. The parts per million (ppm) of ammonia in the sample was determined by colorimetric analysis of the Nessler reaction product at 420 nm^81^. Molarity (M) of ammonia was calculated by conversion of ppm to molarity using the molar mass of ammonia (Eq. 6)

6 $$\:M=\frac{(ppm/1000\:)}{17.031\:g/mol}$$

### Transmission electron microscopy

An overnight culture of *H. meridiana* was pelleted, resuspended and exposed for 2 h to the following solutions: unamended yeast media (control), 1 M ammonia and a pH-matched solution (pH 10.78). The matched pH solution was created by gradual addition of 1 M NaOH to yeast media. Following incubation, cells were washed and resuspended in phosphate buffered saline (PBS). Samples were fixed in 3% glutaraldehyde in 0.1 M Sodium Cacodylate buffer, pH 7.3, for 2 h then washed in three 10-minute changes of 0.1 M Sodium Cacodylate. Specimens were then post-fixed in 1% Osmium Tetroxide in 0.1 M Sodium Cacodylate for 45 min, then washed in three 10-minute changes of 0.1 M Sodium Cacodylate buffer. These samples were then dehydrated in 50%, 70%, 90% and 100% ethanol (X3) for 15 min each, then in two 10-minute changes in Propylene Oxide. Samples were then embedded in TAAB 812 resin. Sections, 1 μm thick were cut on a Leica Ultracut ultramicrotome, stained with Toluidine Blue, and viewed in a light microscope to select suitable areas for investigation. Ultrathin sections, 60 nm thick were cut from selected areas, stained in Uranyl Acetate and Lead Citrate then viewed in a JEOL JEM-1400 Plus TEM. Representative images were collected on a GATAN OneView camera at 4 K resolution. Images were processed using ImageJ^58^.

### Metabolomics sampling and extraction

An overnight culture of *H. meridiana* was inoculated to OD_600_ = 0.05 within 0.25 M ammonia, pH-matched yeast media (pH 10.18) or unamended yeast media (control) within a 24-well plate. The pH-matched solution was created by gradual addition of 1 M NaOH to yeast media. Growth occurred within plates at 28 ºC and was assessed by OD_600_ reading every half an hour using a BMG SPECTROstar Nano Microplate Reader. Samples were harvested during the log phase of growth at OD_600_ = 0.5. All the following procedures occurred at 4 °C. Samples were aliquoted into microcentrifuge tubes and quenched by rapid cooling through submersion in a dry ice/70% (v/v) ethanol bath after brief incubation on ice. During cooling, samples were mixed vigorously to prevent freezing. Cells were separated from spent medium by centrifugation at 1,000 *g* for 10 min and supernatant discarded. Metabolite extraction occurred by addition of ice-cold chloroform/methanol/water (1:3:1 ratio). Cell lysis was encouraged by sonication with ice for 5 min at 37 kHz within an ultrasonication bath (Elmasonic S 60 H). Extraction mixtures were mixed vigorously at 1,200 rpm on an orbital shaker for 1 h. Following mixing, samples were centrifuged at 13,000 *g* for 3 min. The supernatant, containing metabolites, was collected into sterile microcentrifuge tubes. A pooled quality control sample was generated by combining equal volumes of metabolites from each sample. All samples were stored at − 80 °C until analysis.

### Untargeted metabolomics analysis

The untargeted metabolomics analysis was performed using liquid chromatography (LC) coupled to ion mobility (IM) quadrupole time-of-flight (qTOF) mass spectrometry (MS). The instrumentation consisted of an Agilent 1290 Infinity II series UHPLC system hyphenated with an Agilent 6560 IM-qTOF with a Dual Agilent Jet Stream Electron Ionization source. LC separation was performed on an InfinityLab Poroshell 120 HILIC-Z, 2.1 mm × 50 mm, 2.7 μm UHPLC column (Agilent Technologies 689775–924) coupled to an InfinityLab Poroshell 120 HILIC-Z, 3.0 mm × 2.7 μm UHPLC guard column (Agilent Technologies 823750–948). A 3.5 min gradient was run using organic buffer (acetonitrile) combined with an aqueous buffer with low pH (10 mM ammonium formate, pH 3) or high pH (10 mM ammonium acetate, pH 9) for positive and negative ionization modes, respectively. Data was acquired using MassHunter Data Acquisition 10.0 software on 1 µL of sample separated on the column with a flow rate of 800 µL/min. The quality control sample was injected five times at the beginning of the experiment to condition the column and after every five test samples to monitor the instrument state throughout data acquisition. Data were acquired in the 50 to 1700 m/z range, with an MS acquisition rate of 0.8 scans/s. The metabolomics analyses were carried out by the EdinOmics research facility (RRID: SCR_021838) at the University of Edinburgh.

### Metabolomics data processing and statistical analysis

The raw data files were processed using the Agilent MassHunter software suite. Briefly, ion multiplexed data files and calibration files were demultiplexed using the PNNL PreProcessor v2020.03.23 (the default settings for demultiplexing, moving average smoothing, saturation repair and spike removal were applied to the data). The data files were recalibrated for accurate mass and drift time using the AgtTofReprocessUi and the IM-MS Browser 10.0, respectively. Molecular features were extracted in Mass Profiler 10.0 with a retention time tolerance of ± 0.3 min, drift time tolerance of ± 1.5% and accurate mass tolerance of ± (5 ppm + 2 mDa). Features were annotated based on accurate mass and collision cross section (CCS) values using McLean CCS Compendium PCDL library^82^. Multivariate and univariate statistical analysis was performed using the MetaboAnalyst 6.0 web-based platform^83^. The input data were log-transformed and Pareto-scaled. The results were visualised using principal component analysis (PCA), box and whisker plots, volcano plots and heatmaps. Further aesthetic adjustments, including font changes and line thickness modifications, were made using Inkscape (version 1.0.1). Chemical structures were drawn using ChemDraw (version 23.1.2) and exported in SVG format for inclusion in the figures. The raw data associated with this dataset is available to view in the Supplementary Dataset. This study focuses on metabolomic changes in ammonia, pH and control samples, but the broader metabolomics profiling also included samples exposed to (NH_4_)_2_SO_4_. For analysis, only ammonia, pH and control samples were included in this study. The metabolomics of (NH_4_)_2_SO_4_ exposed samples are to be addressed in a separate analysis.

### Statistics and reproducibility

All data were compiled from a minimum of three different cultures inoculated on different days with new media (*n* = 3–4 biological replicates). The normality of data was assessed with the Shapiro-Wilk test. For comparison of two groups, unpaired two-tailed t-tests were used. The Mann Whitney U test was used for unpaired groups where the assumption of normality was violated. An unpaired t-test with Welch’s correction was applied for groups with unequal variance as assessed by an F test. For comparison of two or more groups, equal variance was assessed with the Brown-Forsythe test. Samples of equal variance were examined by analysis of variance (ANOVA) followed by Tukey’s post-hoc test. For samples where variance was not equal, Welch’s ANOVA test with Games-Howell’s post-hoc test was used. Statistical tests are specified in figure legends. Significance was considered when *p* < 0.05. All statistical analyses were performed using GraphPad Prism version 8.0.2 (GraphPad Software Inc.).

## Electronic supplementary material

Below is the link to the electronic supplementary material.

Supplementary Material 1

Supplementary Material 2

## Data Availability

All data generated or analysed during this study are included in this published article within the Supplementary Dataset, except when specified as part of the Supplementary Material.
