# Author Correction: Ammonia sets limit to life and alters physiology independently of pH in *Halomonas meridiana*

**DOI:** 10.1038/s41598-025-26844-x

**Published:** 2025-11-12

**Authors:** Cassie M. Hopton, Peter Nienow, Charles S. Cockell

**Affiliations:** 1 UK Centre for Astrobiology, School of Physics and Astronomy, University of Edinburgh, James Clerk Maxwell Building, Peter Guthrie Tait Road, Edinburgh, EH9 3FD UK; 2 School of Geosciences, University of Edinburgh, Drummond St, Edinburgh, EH8 9XP UK

Correction to: *Scientific Reports* 10.1038/s41598-025-03858-z, published online 04 June 2025.

The original version of this Article was revised to correct outdated information.

Firstly, in the Introduction, the statement:

“The small size and non-polar properties of NH_3_ facilitates passive permeation through membranes^12,13,14,15^, causing significant intracellular disruption^16,17,18^.”

now reads:

“The small size and unchanged nature of NH_3_ facilitates passive permeation through membranes^12,13,14,15^, causing significant intracellular disruption^16,17,18^.”

And where:

“With a pH predicted at or above 9^29,30^, the ocean of Enceladus would bear appreciable amounts of toxic NH_3_.”

now reads:

“With a pH predicted at or above 9^29,30,31^, the ocean of Enceladus would bear appreciable amounts of toxic NH_3_.”

Furthermore, in the Results section, under the subheading ‘Ammonia exposure elicits a unique metabolomic response’, where:

 “Multivariate analysis also revealed exposure to 0.25 M ammonia increased the relative abundance of pantothenate (F = 28.839, *p* < 0.001) (Fig. 6c), and amino acids D-allo-isoleucine (F = 28.686, *p* < 0.001) (Fig. 6d), and alanine (F = 25.391, *p* < 0.01) (Fig. 6e), compared to those in NaOH pH 10.18 and control conditions.”

now reads:

“Univariate analysis also revealed exposure to 0.25 M ammonia increased the relative abundance of pantothenate (F = 28.839, *p* < 0.001) (Fig. 6c), and amino acids D-allo-isoleucine (F = 28.686, *p* < 0.001) (Fig. 6d), and alanine (F = 25.391, *p* < 0.01) (Fig. 6e), compared to those in NaOH pH 10.18 and control conditions.”

In addition, under the Materials and Methods section, under the subheading ‘Bacterial culture’, where:

“Thus, *H. meridiana* Slthf1 was grown in a yeast media consisting of 1 g/100 mL Bacto™ yeast extract (Becton, Dickinson and Company), 0.2 M NaCl (1.17% salinity) (Thermo Fisher Scientific, CAS Number: 7647-14-5) and deionised water (dH_2_O).”

now reads,

“Thus, *H. meridiana* Slthf1 was grown in a yeast media consisting of 1 g/100 mL Bacto™ yeast extract (Becton, Dickinson and Company), 0.2 M NaCl (1.17% salinity) (Thermo Fisher Scientific, CAS Number: 7647-14-5) and distilled water (dH_2_O).”

And Eq. 3, where:

3 $$NH_{3} = NH_{3} + NH_{4}^{ + } /(1 + 10^{{\left( {pKa^{s} + 0.0324\left( {298 - T} \right) + 0.0415\left( P \right)/T - 1} \right)}}$$

now reads,

3 $$\% NH_{3} = 100/\left[ {1 + 10^{{\left( {pKa^{s} + 0.0324\left( {298 - T} \right) + 0.0415\left( {P/T} \right) - pH} \right)}} } \right]$$

In the Discussion section, where:

Interpreted from the point of view of planetary habitability, the closed-air system is the most applicable representation of icy moon subsurface oceans as ammonia concentrations are presumed to remain constant. The ammonia toxicity limit at 0.05 M established in the closed-air system is higher than the lowest predicted concentration of ammonia at 0.01 M for the Enceladus ocean, but lower than the highest predicted concentration at 0.1 M^25,30^. With oceanic temperatures estimated at 0 ºC^28^ and salinity at 4% ^29^, the Enceladus ocean would require a pH of < 9.96 to satisfy a relative abundance of NH_3_ at < 62% suggested as a habitability boundary in this study, which is in the range of current estimations^29,30^.

now reads,

Interpreted from the point of view of planetary habitability, the closed-air system is the most applicable representation of icy moon subsurface oceans, as ammonia concentrations are presumed to remain constant. The ammonia toxicity limit at 0.05 M established in the closed-air system is higher than the lowest predicted concentration of ammonia at 0.01 M for the Enceladus ocean, but lower than the highest predicted concentration at 0.1 M^25,30,31^. When considering the proportion of more toxic NH_3_, the pKa of the NH_3_/NH_4_^+^ system can be presumed to increase as temperature decreases; a pKa of ~ 10.1 at 0 °C has been approximated^32^. The pKa of the NH_3_/NH_4_^+^ equilibrium is also influenced by ionic strength^33^. Thus, with oceanic temperatures estimated at 0 ºC^28^ and ionic strength at 0.333 molal^31^, it can be calculated that the Enceladus ocean would require a pH of ≤ 10.6 to satisfy a relative abundance of NH_3_ at < 62% suggested as a habitability boundary in this study. This is in the range of the lower pH estimations currently modelled for Enceladus, where phosphate speciation in the waters constrains the ocean to pH 10.1 to 11.6, with a most consistent value of pH 10.6 ^31^."

And finally, the following References have been included:

31. Glein, C. R. & Truong, N. Phosphates reveal high pH ocean water on Enceladus. Icarus 441, 116,717 (2025).

32. Bates, R. G. & Pinching, G. D. Acidic dissociation constant of ammonium ion at 0 to 50 °C, and the base strength of ammonia. J. Res. Natl. Bur. Stan. 42, 419 (1949).

33. Bower, C. E. & Bidwell, J. P. Ionization of ammonia in seawater: effects of temperature, pH, and salinity. J. Fish. Res. Bd. Can. 35, 1012–1016 (1978).

The original version of this Article has been corrected.

