# Supplementary material for: Ammonia sets limit to life and alters physiology independently of pH in Halomonas meridiana
Source: Sci Rep. 2025 Jun 4;15:19549. doi: 10.1038/s41598-025-03858-z (PMC12137954; doi:10.1038/s41598-025-03858-z)
Supplement: Supplementary file 2 — Supplementary Material 2 [file 41598_2025_3858_MOESM2_ESM.pdf]

Supplementary Figure 1: pKa^s of NH_4^+ at given ionic strengths. Scatter plot shows the relationship between the stoichiometric acid hydrolysis constant (pKa^s) of NH_4^+ at increasing ionic strengths (I). A linear regression (dotted line) was fitted to the data.

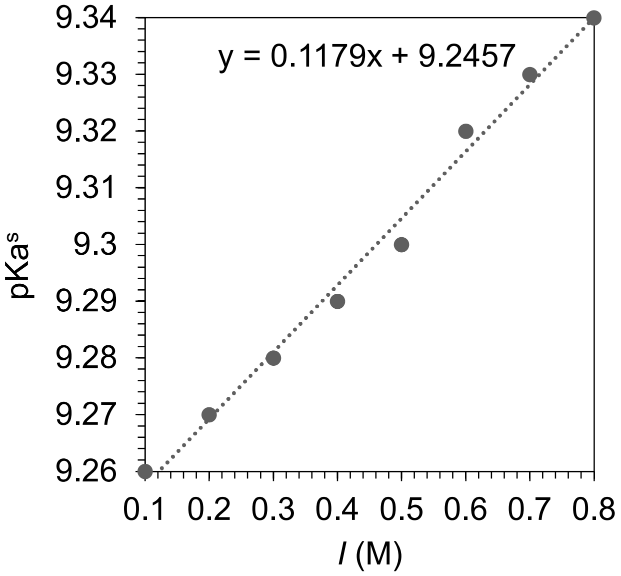

Supplementary Figure 2: Cell viability and optical density at 600 nm over 24 h. Marker and line plot show the PrestoBlue™ cell viability (closed black circles) against optical density at 600 nm (OD_{600}) readings (open white squares). Markers present the mean ± s.d. (n =3). The s.d. is smaller than the marker if error bar is not visible

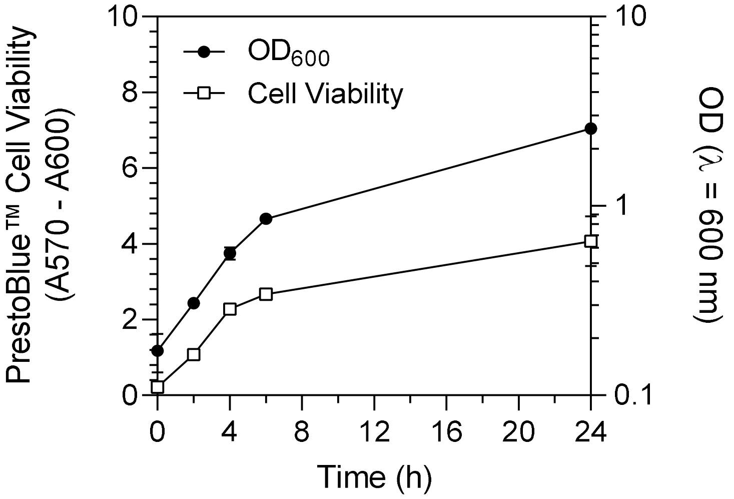

Supplementary Table 1: Outcome of statistical analysis by unpaired t-test and Mann-Whitney test comparing the oxygen measurements (Fig. 1c) and water activity (Fig. 1d) for ammonia and NaOH solutions at each pH value. The use of Mann-Whitney is specified by ^{MW}. All other tests are unpaired t-test. ns, no significance; *, $p < 0.05$; **, $p < 0.01$; ***, $p < 0.001$; ****, $p < 0.0001$.

Oxygen measurements

NaOH - ammonia	Significance	Test outcome
pH 8.05	ns	t=1.8, df=6, p = 0.122
pH 8.96	ns	t=0.150, df=6, p = 0.886
pH 9.38	ns	t=0.203, df=6, p = 0.846
pH 9.73	ns	t=0.257, df=6, p = 0.806
pH 10.18 ^{MW}	ns ^{MW}	U=4, p = 0.343 ^{MW}
pH 10.49	ns	t=2.053, df=6, p = 0.0859
pH 10.78	ns	t=0.272, df=6, p = 0.795

Water activity

NaOH - ammonia	Significance	Unpaired t-test outcome
pH 8.05	ns	t=0.768, df=6, p = 0.472
pH 8.96	ns	t=0.247, df=6, p = 0.813
pH 9.38	ns	t=0.396, df=6, p = 0.706
pH 9.73	ns	t=0.424, df=6, p = 0.686
pH 10.18	ns	t=0.533, df=6, p = 0.613
pH 10.49	ns	t=1.093, df=6, p = 0.316
pH 10.78	ns	t=0.415, df=6, p = 0.693

Supplementary Table 2: Statistical outcomes for one-way ANOVA with Tukey's post-hoc test comparing the mean cell number (CFU/mL) of *H. meridiana* grown in positive control (PC) solutions (0 M ammonia, no pH modification) and NaOH solutions pH-matched to ammonia solutions (Fig. 3a). ns, no significance; *, $p < 0.05$; **, $p < 0.01$; ***, $p < 0.001$; ****, $p < 0.0001$.

Comparison groups	Significance	*p*-value
PC - pH 8.05	ns	>0.9999
PC - pH 8.96	ns	0.4384
PC - pH 9.38	ns	0.9999
PC - pH 9.73	ns	0.9961
PC - pH 10.18	*	0.0410
PC - pH 10.49	ns	0.7936
PC - pH 10.78	ns	0.8097

Supplementary Table 3: Statistical tests utilised for the comparison of the data for lag phase (Fig. 3c), doubling time (*T*d) (Fig. 3d) and Final OD (Fig. 3e) of *H. meridiana* grown in ammonia vs. pH-matched NaOH solutions.

NaOH - ammonia	Lag Phase	*T*d	Final OD
pH 8.05	Mann-Whitney t-test,	Unpaired t-test	Unpaired t-test
pH 8.96	Unpaired t-test with Welch's correction	Unpaired t-test	Unpaired t-test with Welch's correction
pH 9.38	Unpaired t-test with Welch's correction	Unpaired t-test	Unpaired t-test
pH 9.73	Unpaired t-test with Welch's correction	Unpaired t-test	Unpaired t-test
pH 10.18	Unpaired t-test with Welch's correction	Mann-Whitney t-test,	Unpaired t-test
pH 10.49	n/a	n/a	Unpaired t-test with Welch's correction
pH 10.78	n/a	n/a	Unpaired t-test with Welch's correction

Supplementary Table 4: Volcano analysis comparing molecular features in the 0.25 M ammonia dataset against the control dataset (n=3) (Fig. 5c), where metabolites identified in the analysis exhibited a fold change (FC) greater than 2 and a *p*-value < 0.05 (adjusted using FDR correction). Volcano analysis was performed using the web-based software MetaboAnalyst 6.0.

Metabolite	FC	log2(FC)	p.ajusted	-log10(p)
CMP-Sialic acid	3.86E+07	25.202	1.20E-07	6.9196
9-OxoODE	1.89E-08	-25.654	2.79E-06	5.5541
N-Acetylserotonin	8.06E-08	-23.565	2.98E-06	5.5258
Glutarylcarnitine	1.23E-09	-29.595	1.62E-05	4.7898
Mevalonolactone	4.82E+08	28.845	1.62E-05	4.7898
Linolenic Acid	1.49E-08	-26	1.62E-05	4.7898
2,7-Dimethylnaphthalene	3.45E+07	25.038	1.68E-05	4.7747
Shikimic Acid	3.42E-07	-21.481	1.68E-05	4.7747
Glycerol-3-phosphate	8.34E+07	26.313	2.20E-05	4.6574
Bishomo-gamma-linolenic Acid (20:3)	2.43E-08	-25.293	2.47E-05	4.607
Indole-3-ethanol	1.72E-09	-29.115	5.33E-05	4.2733
Atrazine	4.19E+06	21.997	5.93E-05	4.2268
PA 36:02	5.80E+08	29.113	6.34E-05	4.1982
Sparfloxacin	1.39E+08	27.049	6.47E-05	4.1893
5-Hydroxyindoleacetate	4.53E+08	28.754	0.0002	3.6996
PS 37:04	2.48E+08	27.886	0.000412	3.385
PS 40:01	3.16E-07	-21.592	0.00063	3.2005
PE 38:02	8.20E-08	-23.539	0.002132	2.6712
GVLHAVK (Tryptic Peptide)	2.51E+07	24.582	0.002461	2.6088
D-allo-Isoleucine	3.79E+00	1.9222	0.020352	1.6914
Sorbitol	1.11E-01	-3.1706	0.039119	1.4076
Pantothenate	2.65E+00	1.4068	0.039119	1.4076
Alanine	2.89E+00	1.5335	0.045708	1.34

Supplementary Table 5: Volcano analysis comparing molecular features in the 0.25 M ammonia dataset against those in the NaOH pH 10.18 dataset (n=3) (Fig. 5d), where metabolites identified in the analysis exhibited a fold change (FC) greater than 2 and a *p*-value < 0.05 (adjusted using FDR correction). Volcano analysis was performed using the web-based software MetaboAnalyst 6.0.

Metabolite	FC	log2(FC)	p.ajusted	-log10(p)
ELR (Tryptic Peptide)	5.11E+08	28.928	2.52E-05	4.5991
2,7-Dimethylnaphthalene	3.45E+07	25.038	6.44E-05	4.191
Uric Acid	2.01E-07	-22.248	0.000134	3.8726
Atrazine	4.19E+06	21.997	0.000171	3.7681
3-Hydroxybenzaldehyde	1.97E-08	-25.597	0.000191	3.7186
PC (16:1/16:1) (del9-cis)	1.06E-07	-23.172	0.000191	3.7186
Tributylammonium	3.59E-07	-21.411	0.000191	3.7186
Astilbin	7.94E-08	-23.586	0.0002	3.6995
allo-Threonine	1.71E+07	24.03	0.000287	3.5424
3,4-Dihydroxy-L-phenylalanine	4.74E+06	22.175	0.000912	3.0399

Supplementary Table 6: Volcano analysis comparing molecular features in the NaOH pH 10.18 dataset against those in the control dataset (n=3) (Fig. 5e), where metabolites identified in the analysis exhibited a fold change (FC) greater than 2 and a *p*-value < 0.05 (adjusted using FDR correction). Volcano analysis was performed using the web-based software MetaboAnalyst 6.0.

Metabolite	FC	log2(FC)	p.ajusted	-log10(p)
PS 37:04	1.07E+09	29.99	5.76E-11	10.24
Fumarate	3.92E+08	28.546	2.03E-06	5.693
9-OxoODE	1.89E-08	-25.654	2.03E-06	5.693
N-Acetylserotonin	8.06E-08	-23.565	2.19E-06	5.6599
PS 41:06	1.80E+07	24.099	6.28E-06	5.202
1,2-Dimyristoyl-sn-glycero-3-phosphocholine	1.24E-07	-22.941	6.28E-06	5.202
NVNDVIAPAFVK (Tryptic Peptide)	3.55E-09	-28.07	1.02E-05	4.9923
Linolenic Acid	1.49E-08	-26	1.02E-05	4.9923
Glutarylcarnitine	1.23E-09	-29.595	1.06E-05	4.975
Adenosine-5'-monophosphate	8.12E-10	-30.198	1.09E-05	4.9635
Glycerol-3-phosphate	8.37E+07	26.319	1.68E-05	4.7746
5-Hydroxymethyluracil	1.65E-08	-25.852	1.68E-05	4.7746
ELR (Tryptic Peptide)	2.11E-09	-28.821	1.72E-05	4.7637
Bishomo-gamma-linolenic Acid (20:3)	2.43E-08	-25.293	1.72E-05	4.7637
Reserpine	3.77E+07	25.167	1.72E-05	4.7637
Indole-3-ethanol	1.72E-09	-29.115	3.59E-05	4.4451
allo-Threonine	6.04E-08	-23.981	4.15E-05	4.3815
CMP-Sialic acid	1.75E+07	24.058	4.37E-05	4.3599
Thiabendazole	6.62E+06	22.659	4.37E-05	4.3599
Terbutryn	4.61E+06	22.137	7.12E-05	4.1475
Imazapyr	3.46E-08	-24.784	7.31E-05	4.136
Astilbin	1.26E+07	23.586	7.42E-05	4.1295
N-(3-Phenylpropionyl)-glycine	9.76E-08	-23.288	0.000154	3.8122
PE 38:02	8.20E-08	-23.539	0.001566	2.8052
PE (O-34:03)	5.53E+01	5.788	0.022256	1.6526
N-Acetylglutamate	2.24E+00	1.1659	0.042834	1.3682
Riboflavin	4.18E-01	-1.259	0.04393	1.3572
LysoPC (13:0)	3.92E+00	1.9699	0.049445	1.3059

Supplementary Table 7: Metabolites significantly altered (*p*-value lower than 0.05 (FDR corrected) between the three treatment conditions assessed by ANOVA with Tukey's multiple comparison test (n=3) (Fig. 5f). *p*-values without FDR adjustment are also shown. ANOVA analysis was performed using the web-based software MetaboAnalyst 6.0.

Metabolite	f.value	p.value	-log10(p)	FDR	Tukey's HSD
9-OxoODE	23884	1.98E-12	11.703	1.11E-09	Ammonia-Control; PH-Control
N-Acetylserotonin	18876	4.01E-12	11.397	1.12E-09	Ammonia-Control; PH-Control
Linolenic Acid	6186.4	1.1387E-10	9.9436	2.0122E-08	Ammonia-Control; PH-Control
Glutarylcarnitine	5717.4	1.4424E-10	9.8409	2.0122E-08	Ammonia-Control; PH-Control
2,7-Dimethylnaphthalene	4865.6	2.34E-10	9.6309	2.61E-08	Ammonia-Control; PH-NH3
Bishomo-gamma-linolenic Acid (20:3)	3586.7	5.84E-10	9.2338	5.43E-08	Ammonia-Control; PH-Control
ELR (Tryptic Peptide)	2697.2	1.37E-09	8.8628	1.09E-07	PH-Control; PH-Ammonia
Indole-3-ethanol	2327.8	2.13E-09	8.6712	1.49E-07	Ammonia -Control; PH-Control
Atrazine	2112.3	2.8525E-09	8.5448	1.6497E-07	Ammonia -Control; PH- Ammonia
Glycerol-3-phosphate	2022.2	3.25E-09	8.488	1.65E-07	Ammonia -Control; PH-Control
CMP-Sialic acid	2021.9	3.25E-09	8.4878	1.65E-07	Ammonia -Control; PH-Control
Astilbin	1379.1	1.02E-08	7.9902	4.76E-07	PH-Control; PH-Ammonia
PS 37:04	765.86	5.9404E-08	7.2262	2.5498E-06	Ammonia -Control; PH-Control; PH-Ammonia
allo-Threonine	715.65	7.27E-08	7.1382	2.90E-06	PH-Control; PH-Ammonia
PE 38:02	284.79	1.1328E-06	5.9458	0.00004214	Ammonia -Control; PH-Control
PE (O-34:03)	45.702	0.00023373	3.6313	0.0081513	Ammonia -Control; PH-Control
Sorbitol	32.74	0.00059145	3.2281	0.019413	Ammonia -Control; PH-Control
N,N-Dimethylglycine	30.626	0.00071015	3.1487	0.022015	Ammonia -Control; PH-Control

Pantothenate	28.839	0.00083655	3.0775	0.022551	Ammonia -Control; PH- Ammonia
PC 34:02	28.822	0.000838	3.0768	2.26E-02	Ammonia -Control; PH-Control
D-allo-Isoleucine	28.686	0.000849	3.0712	2.26E-02	Ammonia -Control; PH-NH3
N-Acetylglutamate	25.653	0.0011478	2.9401	0.028623	Ammonia -Control; PH-Control
Alanine	25.391	0.0011798	2.9282	0.028623	Ammonia -Control; PH-NH3
N-Acetyl-L-aspartic Acid	24.091	0.0013579	2.8671	0.031572	Ammonia -Control; PH-Control
Stearic Acid (18:0)	22.984	0.001539	2.8128	0.033617	Ammonia -Control; PH-Control
Indoleacetaldehyde	22.832	0.0015664	2.8051	0.033617	Ammonia -Control; PH- Ammonia
Mannitol	20.965	0.0019616	2.7074	0.04054	Ammonia -Control; PH-Control
Riboflavin	19.872	0.0022566	2.6465	0.044971	PH-Control; PH-Ammonia
L-Tryptophanamide	19.588	0.0023426	2.6303	0.045076	Ammonia -Control; PH-Control
PE 36:05	18.741	0.0026273	2.5805	0.048867	Ammonia -Control; PH-Control

Supplementary Table 8: Relative abundance of NH_3 was calculated from equations Eq.1.0, 2.0 and 3.0 depicted in the main text. Table shows parameters and values for salinity (S), temperature (T), ionic strength (I), pKa^s and % NH_3 calculated. The pKa^s was calculated from the linear regression provided by Supplementary Figure 1.

Am (M)	Am (mg/L)	S (ppt)	pH	T (K)	I	pKa^s	NH_3 (mg/L)	% NH_3	% NH_4^+
0.010	170.31	11.688	8.05	301.15	0.236	9.274	11.96	7.0	93.0
0.025	425.78	11.688	8.96	301.15	0.236	9.274	161.97	38.0	62.0
0.050	851.55	11.688	9.38	301.15	0.236	9.274	525.89	61.8	38.2
0.100	1703.1	11.688	9.73	301.15	0.236	9.274	1334.08	78.3	21.7
0.250	4257.75	11.688	10.18	301.15	0.236	9.274	3877.22	91.1	8.9
0.500	8515.5	11.688	10.49	301.15	0.236	9.274	8124.94	95.4	4.6
1.000	17031	11.688	10.78	301.15	0.236	9.274	16621.2	97.6	2.4
